# Comparison Between Computed Tomography Arthrography (CTA) Without Traction, CTA With Traction, and Magnetic Resonance Arthrography in Hip Impingement: What Is the Best Method?

**DOI:** 10.7759/cureus.84813

**Published:** 2025-05-26

**Authors:** Benjamin Dallaudiere, Lionel Pesquer, Caroline Ziade, Pierre Abadie, Philippe Meyer, Pierre Francois Lintingre

**Affiliations:** 1 Radiology, Clinique du Sport de Bordeaux Mérignac, Bordeaux, FRA; 2 Orthopaedic Surgery, Clinique du Sport de Bordeaux Mérignac, Bordeaux, FRA

**Keywords:** 3d ct scan, articular cartilage, hip joint, magnetic resonance arthrography, superior labrum

## Abstract

Purpose: Femoro-acetabular impingement (FAI) is a possible mechanism for the development of early osteoarthritis. FAI occurs when there is a conflict between the rim of the acetabulum and the femoral neck, usually due to irregularities of the proximal femur or acetabulum. Our aims were to compare the diagnostic efficacy of computed tomography arthrography (CTA) without traction, CTA with traction, and magnetic resonance arthrography (MRA) for the detection and grading of chondral and labral lesions in patients with a clinically positive impingement test and to determine the correlation between imaging findings and arthroscopic data in patients who had undergone surgery.

Patients and methods: This monocentric, observational, retrospective study was conducted on 95 consecutive patients referred to our imaging department with clinical symptoms suggestive of FAI between January 2012 and May 2023. A total of 21 patients underwent therapeutic arthroscopy after the failure of medical treatment. These patients underwent either CTA without traction, CTA with traction, or MRA. Chondral and labral lesions were assessed by two musculoskeletal radiologists who were unaware of the original arthroscopy findings and interpretations. The modified MAHORN (Multicenter Arthroscopy of the Hip Outcomes Research Network) classification was used for grading during imaging and surgery.

Results: There was no significant difference between the examination techniques for the diagnosis of labral lesions (p=0.1737). Cartilage lesions preferentially affected the acetabular side compared to the femoral side, with no significant difference between the examination techniques for the diagnosis of chondral lesions (p=0.1429 for femoral cartilage, p=0.0944 for acetabular cartilage). No significant difference was found between the imaging and surgical data for the diagnosis of labral and cartilage lesions, even when the three imaging techniques were combined (labrum, p=0.1797; acetabular cartilage, p=0.0588; femoral cartilage, p=0.6547).

Conclusion: No significant difference was found between CTA without traction, CTA with traction, and MRA procedures for the detection and grading of chondral and labral lesions. Furthermore, there was no significant difference between the imaging and surgical data when the three imaging techniques were combined.

## Introduction

Hip osteoarthritis (OA) is a multifactorial pathology caused by environmental, biomechanical, and genetic factors. When it occurs in young adults, a morphological disorder such as dysplasia, protrusio acetabuli, neck fracture sequelae, history of slipped capital femoral epiphysis, or Legg-Perthes-Calvé disease must be investigated. For most nondysplastic hips, femoro-acetabular impingement (FAI) has recently been identified as a possible mechanism for the development of early OA [1]. FAI occurs as a result of conflict between the rim of the acetabulum and the femoral neck due to deformities in the proximal femur or acetabulum, which have two different causes. Cam-type impingement is due to an aspherical junction between the head and neck and a reduced depth of the femoral waist. It is represented by an osseous bump at the femoral neck, which causes damage to the cartilage around the anterosuperior rim [2]. In contrast, pincer-type impingement is due to a deep acetabulum and large posteroinferior cartilage lesions [3].

Visualization of cartilage degeneration and a labral defect in FAI by imaging is fundamental and can lead to surgical treatment with the goal of delaying hip replacement [4]. The aim of hip preservation surgery (arthroscopic or open) is to relieve pain and improve hip function by treating the structural lesions causing FAI [5]. The strongest predictor of a poor outcome after surgery for FAI is the presence of pre-existing advanced OA [6,7]. In our opinion, the challenge of hip imaging is to expose the articular cartilage layer, labral-chondral interface, and ligamentum teres to avoid diagnostic arthroscopy and adapt treatment [8].

Computed tomography arthrography (CTA) has been reported to be an effective technique to detect labral tears, providing results that correlate well with arthroscopy findings (sensitivity and specificity of ~90%) in patients with FAI [9]. The hip is a close-fitting, ball-and-socket joint lined with articular cartilage. The implementation of traction during CTA is a simple and inexpensive way to widen the intra-articular joint space and thus reveal both the acetabular and femoral cartilage [10]. However, there is no consensus on the amount of traction necessary, with various reports describing traction ranging from 6 to 25 kg; the amount of traction applied will depend on the weight of the patient, how well they are able to support traction, and the hip morphology [10-13]. Patients with normal hips or with acetabular retroversion and FAI are likely to require and support greater traction than individuals with hip dysplasia [14]. However, traction usually results in pain during and after the procedure [14].

In comparison to CTA, Naraghi and White [15] carried out a review of 19 studies and reported that magnetic resonance arthrography (MRA) had a sensitivity of 69-100% for the detection of labral tears, with 12 studies reporting a sensitivity of >90% [15]. MRA is a non-irradiating technique and is, therefore, attractive for young people. However, it requires a long examination time, has a relatively low spatial resolution, rendering the detection of very small or thin structures difficult, and is costly.

To our knowledge, there have been no previous reports comparing CTA with traction, CTA without traction, and MRA without traction for the detection of chondral and labral lesions or the correlation between these three methods and clinical gold standard data.

This study aimed to compare the diagnostic efficacy of these three procedures for the detection and grading of chondral and labral lesions in patients with a clinically positive impingement test and to evaluate the correlation between imaging findings and arthroscopic data in patients who had undergone surgery.

## Materials and methods

Patients

This monocentric, observational, retrospective study was carried out on 95 consecutive patients referred to our imaging department with clinical symptoms suggestive of FAI between January 2012 and May 2023. Patients were included in the study if they had hip pain, typically localized to the groin, limitation of range of motion, or clicking, identified by an orthopedic surgeon. Teenagers <18 years old were also included. The symptoms could be exacerbated by physical activity. The test in flexion, adduction, and medial rotation was usually positive. The physical examination eliminated other differential diagnoses: fascia lata or psoas flap, nerve compression (femoral and sciatic nerve), and adductor tendon pain. Patients were excluded from the study if they had a contraindication to magnetic resonance imaging (MRI) or an allergy to iodinated contrast agents.

At inclusion, all patients underwent a pelvic X-ray with a side profile of the neck of the femur in order to evaluate femoro-acetabular narrowing and hip morphology [16]. The initial X-rays could show hip OA. Patients then underwent either CTA without traction, CTA with traction, or MRA. The study was conducted in accordance with the ethics and principles of the Declaration of Helsinki.

Imaging technique

MRA Protocol

Prior to MRA, the patients received an anterolateral, intra-articular injection containing 1 mL lidocaine hydrochloride (Xylocaine®, 10 mg/mL; AstraZeneca, UK), 1 mL iodinated contrast agent, iodixanol (Visipaque®, 270 mg/mL; GE Healthcare, USA), and 10 mL of the contrast agent, gadoteric acid (Artirem®, 0.0025 mmol/mL; Guerbet, France). Injections were performed under sterile conditions, with fluoroscopic guidance, using a 21 G needle. Immediately after the injection and five minutes before MRA, the patient was prompted to mobilize their hip to improve the passage of the contrast fluid into the joint.

A 1.5 T unit (Optima MR 450w; GE Healthcare, USA) and coils adapted to the patient's morphology were used with the parameters described in Table 1.

**Table 1:** Parameters of MRA sequences T1-w: T1-weighted sequence; DP FS: density proton-weighted sequence with fat saturation

Sequence	Repetition Time (ms)	Echo Time (ms)	Matrix	Field of View (mm)	Slice Thickness (mm)
Coronal SE T1-w	469	10.2	512 x 384	380	4
Coronal DP FS	2757	58.43	416 x 320	380	3
Axial-oblique T1-w	390	10.5	512 x 320	360	4
Axial-oblique DP FS	3500	56.2	384 x 288	360	4
Sagittal T1-w	475	9.5	416 x 320	280	4

CTA Protocol

Prior to CTA, the patients received an anterolateral, intra-articular injection containing 1 mL lidocaine hydrochloride (Xylocaine®, 10 mg/mL) and 11 mL iodixanol (Visipaque®, 270 mg/mL) or ioxaglate meglumine (Hexabrix®, 320 mg/mL; Guerbet, France). Injections were performed under sterile conditions, with fluoroscopic guidance, using a 21 G needle. As for MRA, the subject was encouraged to mobilize their hip before CTA in order to improve the passage of the contrast fluid into the joint.

Subsequently, the subject was encouraged to walk and mobilize the hip before CTA for about five minutes. Traction was applied, which was made by putting the ankle in a specially manufactured boot to which weights of 16-34 kg were attached, depending on the patient's weight (16, 28, and 34 kg for patients under 55, between 55 and 65, and others, respectively) and the supported traction. The leg was extended caudally with the hip in neutral rotation.

CT images were acquired five minutes after the initiation of traction using a 16-slice machine (Optima CT 580w; GE Healthcare, USA). The scan area was adjusted with a scout image to a field of view centered on the hip. The acquisition parameters were a rotation speed of 5.62 mm/rotation, a current of 350 mAs, a voltage of 140 kV, a collimation of 10 mm, and a slice thickness of 0.625 mm.

Before applying weight, it is crucial to conduct manual axial distraction of the hip joint, enabling continuous traction and ensuring consistent distraction of articular spaces.

Review of images and data analysis

Two musculoskeletal radiologists, with one and five years of specialized experience retrospectively, were asked to come to a consensual agreement on the nature of the chondral and labral lesions revealed by imaging. They were unaware of the original arthroscopy findings and diagnosis and were totally blinded to clinical or radiological data.

Interpretation was performed on a GE Healthcare® post-processing workstation (ADW 4.6). The labral and cartilage lesions observed during imaging and surgery were graded using the modified MAHORN (Multicenter Arthroscopy of the Hip Outcomes Research Network) classification (Table 2) [14-17]. If more than one lesion was observed in the joint, grading was based on the most severe lesion.

**Table 2:** Modified MAHORN classification for the grading of labrum and cartilage lesions CTA: computed tomography arthrography; MRA: magnetic resonance arthrography; MAHORN: Multicenter Arthroscopy of the Hip Outcomes Research Network Table created by the authors

Lesion		Hip Arthroscopy	CTA or MRA
1. Labral-chondral separation		Cleft located at the chondrolabral interface	Focal area of high signal intensity or inflow of contrast located at the chondrolabral interface
2. Partial labral tear		Cleft extending between the labral base and acetabular rim	Hyperintense signal or inflow of contrast extending between the labral base and acetabular rim
3. Complete labral tear		Complete labral avulsion from the acetabulum	Complete interposition of hyperintense signal or contrast between the labral base and acetabular rim
4. Intrasubstance labral tear		Radial/longitudinal cleft within the labral substance	Focal area of hyperintense signal or contrast material extending into the labral surface
5. Complex labral tear		Intersubstance (partial/complete tear) and intrasubstance tear	
6. Bubble	Full-thickness cartilage lesions	Cartilage detached from subchondral bone but intact surface; "carpet phenomenon"	Subchondral line of hyperintense signal with intact cartilage surface
7. Delamination tear		Cartilage delamination with disrupted surface, palpable free edge of the cartilage	Subchondral line of hyperintense signal or inflow of contrast with disrupted cartilage surface
8. Defect		Complete loss of cartilage thickness	Complete loss of cartilage thickness
9. < 50%	Partial-thickness cartilage lesions	Cartilage lesion involving < 50% cartilage thickness	Cartilage lesion involving < 50% cartilage thickness
10. > 50%		Cartilage lesion involving > 50% cartilage thickness	Cartilage lesion involving > 50% cartilage thickness

Joint distraction was measured as the maximum distance between the cartilage surfaces at the proximal point of the femoral head and the acetabulum. Partial volume effects were minimized by measuring joint distraction on coronal and sagittal images.

The alpha angle, which measures the degree to which the femoral head deviates from a spherical shape, was measured as the angle between a line that runs down the axis of the femoral neck and a line from the center of the femoral head to the point where the distance from the femoral head center was greater than the femoral head radius, as described previously [3,18,19].

The thickness of the ligamentum teres was determined on coronal images at a site 5 mm distal to its femoral head insertion [18].

Arthroscopic hip surgery

**Figure 1:**
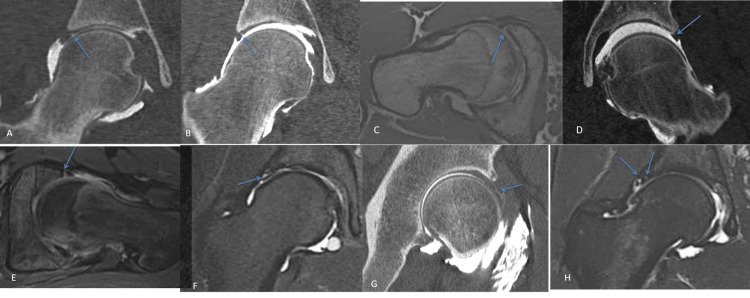
Different kinds of labral lesions (A) Labral-chondral separation in computed tomography arthrography (CTA) without traction and (B) with traction. (C) Partial labral tear in CTA with traction and (D) magnetic resonance arthrography (MRA). (E) Complete labral tear in MRA. (F) Intrasubstance labral tear in MRA and (G) CTA without traction. (H) Complex labral tear in MRA

**Figure 2:**
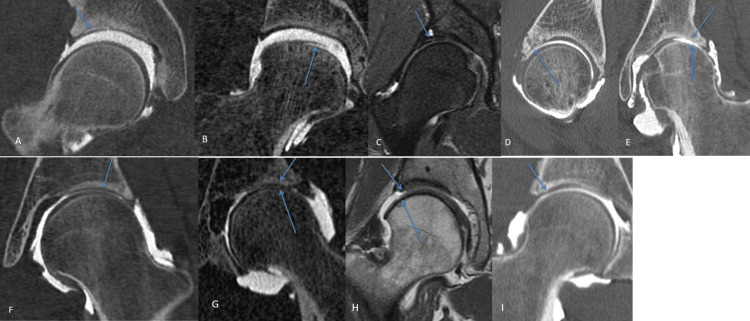
Different kinds of chondral lesions (A) Delamination cartilage lesions in the acetabular or (B) femoral area in computed tomography arthrography (CTA) with traction. (C) Full defect cartilage lesion in magnetic resonance arthrography (MRA) and (D) CTA without traction and (E) with traction. (F) Partial-thickness (<50%) cartilage lesions in CTA without traction and (G) partial-thickness (>50%) cartilage lesions in CTA without traction, (H) MRA, and (I) CTA with traction

In the case of pain resistant to conservative treatment and a preoperative assessment showing signs of impingement without OA, arthroscopic hip surgery could be carried out. An intra-articular checkup was performed after hip disjunction, allowing the visualization, classification, and treatment of chondral and labral lesions (Figures 1, 2). Femoroplasty or acetabuloplasty could be combined.

Data and statistical analysis

All statistical analyses were performed with R software (R Core Team, R Foundation for Statistical Computing, Vienna, Austria). We evaluated the performance of MRA and CTA with and without traction and also compared the results to surgery using a t-test. The significance threshold selected for all the statistical analyses was 0.05. The results from the quantitative variables are presented as mean ± standard deviations (SD) and minimum, maximum, and median values. All comparisons were made using data from surgery as the gold standard.

## Results

Patient characteristics

A total of 95 patients were included in the study (36 women and 59 men). The mean age of the study population was 35.4 years (range: 17-73). In total, 31 patients (32.6%) underwent CTA without traction, 30 (31.6%) had CTA with traction, and 34 underwent MRA (35.8%). The mean ages of the three groups were 39.2, 36.7, and 31 years, respectively. There were 21 patients (22%) who had undergone therapeutic arthroscopy after the failure of medical treatment, including hip injections, pain management with nonsteroidal anti-inflammatory drugs, and physiotherapy.

Joint distraction was obtained in 60% of tractions (18/30) and 9% (6/65) of procedures without traction. The mean (±SD) joint distraction was 4.2 ± 1.8 mm with traction and 3.9 ± 0.9 mm without traction. No disjunction was observed by MRA.

Labral lesions

For the majority of patients in the three groups, the labral lesions were mostly low severity (≤ grade 2) (80% of patients with CTA with traction, 74.3% CTA without traction, 58.9% MRA). Almost one-third of the patients in the CTA without traction (32.3%) and MRA groups (32.4%) had no observable labral lesions (Table 3). There was no significant difference between the examination techniques for the diagnosis of labral lesions (p=0.1737).

**Table 3:** Distribution of grades of labral lesions according to examination techniques CTA: computed tomography arthrography; MRA: magnetic resonance arthrography

Technique	0	1	2	3	4	5
CTA with traction	8	6	10	1	4	1
%	26.7	20	33.3	3.3	13.3	3.3
CTA without traction	10	10	3	4	3	1
%	32.3	32.3	9.7	12.9	9.7	3.2
MRA	11	3	6	7	5	2
%	32.4	8.8	17.7	20.6	14.7	5.9

Chondral lesions

Cartilage lesions preferentially affected the acetabular side compared to the femoral side (Table 4), with no significant difference between the examination techniques for the diagnosis of chondral lesions (p=0.1429 for femoral cartilage, p=0.0944 for acetabular cartilage).

**Table 4:** Distribution of grades of chondral lesions according to examination techniques A: acetabular; F: femoral; CTA: computed tomography arthrography; MRA: magnetic resonance arthrography

Grade	0		7		8		9		10	
	A	F	A	F	A	F	A	F	A	F
CTA with traction	12	24	5	2	5	2	5	1	3	1
%	40	80	16.7	6.7	16.7	6.7	16.7	3.3	10	3.3
CTA without traction	4	17	4	1	9	4	6	6	8	3
%	12.9	54.8	12.9	3.2	29	12.9	19.4	19.4	25.8	9.7
MRA	13	25	1	0	9	3	8	5	3	0
%	38.2	75.8	2.9	0	26.5	9.1	23.5	15.2	8.8	0

Alpha angle

There was no significant correlation between alpha angle and lesion severity. However, 100% of subjects without labral lesions or cartilage lesions (n=13) had an alpha angle of <55°, and 100% of subjects with an alpha angle >55° (n=26) had an impaired labrum and/or cartilage on imaging.

Comparison with arthroscopic hip surgery

Only 21 (22%) patients underwent arthroscopic hip surgery, of which 16 were performed by MRA. No significant difference was found between the imaging and surgical data for the diagnosis of labral and cartilage lesions or when the three imaging techniques were combined (labral lesions, p=0.1737; acetabular cartilage, p=0.0588; femoral cartilage, p=0.6547). The sensitivity (94%) and specificity (88%) of MRA were high. There was a trend towards underestimation of errors in the grading of the lesions.

## Discussion

Our analysis revealed no significant difference between CTA with traction, CTA without traction, and MRA procedures for the detection and grading of chondral and labral lesions, nuancing the fact that traction helps unmask cartilage lesions and confirming a direct correlation between the alpha angle and pathology but without a statistical value (p>0.05) in our series.

It is difficult to interpret hip images in the early stages of OA as the joint space of the hip is very narrow and the initial lesions of the labrum and cartilage are small. In patients with FAI, the challenge of imaging is to detect these lesions as early as possible so that the patient can receive appropriate care, especially if they participate in sports. Therapeutic surgery (open or arthroscopy) makes it possible to assess and repair the labral and cartilaginous lesions (resection of the cartilaginous valves, segmental resection of the labrum, micro-fracturing) and also to correct the cause by femoroplasty. However, surgery is contraindicated in advanced chondropathy because of the inconclusive results in patients with OA.

The specificity of CTA without traction for detecting labral or cartilage involvement is excellent (90-100%). However, its sensitivity decreased to 88% for acetabular cartilage, leading to a high possibility of false negative results [9,20]. Consequently, radiologists have evaluated the addition of traction to open the joint and improve the diagnostic performance of imaging. When traction is not applied, images of the joint are masked by the femoral and acetabular cartilage, whereas opening the joint by traction results in a clearer image and can unmask cartilaginous lesions.

Reports in the literature describe a number of different techniques to apply traction. The current study applied traction using a similar system to that described elsewhere but using slightly heavier weights [21-23]. Henak et al. proposed using different traction methods depending on the hip pathology of their patients. They reported a mean joint distraction after traction of 2.46 mm (vs. 4.2 mm in the current study) [10]. Pain is a common problem during hip examination and may be encountered in over half of the patients during the injection or traction. However, the pain resolves quickly, and the majority of patients are pain-free within 24 hours [14].

The meta-analysis of Smith et al. on the diagnostic performance of MRA for the detection of chondral hip lesions gave disappointing results, with a low sensitivity of 62% in particular (95% CI: 0.57-0.66) and a mean specificity of 86% (95% CI: 0.83-0.89) [24]. The sensitivity calculated in this meta-analysis differs greatly from our acceptable sensitivity of 94%. The small number of patients who had surgery in our cohort may have affected this result. However, our results are similar to those of Schmaranzer et al. in their cohort of 73 patients [14]. Measuring the alpha angle seems to be an additional argument to help in the diagnosis of cam-type impingement, with good interobserver correlation. This can be measured by CT, but also by MRI, with or without traction [23,24].

Surgery may be possible in patients with FAI, but it will depend on the degree of cartilaginous involvement, the patient's age, physical activity, functional improvement in sports practice, and the patient's motivation after being informed about the risks inherent in this kind of surgery.

Our study has some limitations. First, the number of patients is relatively small, but the data are sufficiently uniform to lead to high confidence in the results. Another limitation was the retrospective analysis. However, this model was coherent with the primary goal of this preliminary study, which was to assess CTA without traction, CTA with traction, and MRA to evaluate normal and pathological labral and chondral hip lesions. The next step would be to use a prospective series to confirm our results.

## Conclusions

No significant difference was found between CTA with traction, CTA without traction, and MRA procedures for the detection and grading of chondral and labral lesions. Furthermore, there was no significant difference between imaging and surgical data when the three imaging techniques were combined.
